# Reconstructing goals for transfer of training in faculty development programs for higher education teachers: A qualitative documentary method approach

**DOI:** 10.1016/j.heliyon.2019.e02928

**Published:** 2019-11-28

**Authors:** Andreas Gegenfurtner

**Affiliations:** Technische Hochschule Deggendorf and University of Passau, Germany

**Keywords:** Education, Educational psychology, Pedagogy, Teaching research, Individual differences

## Abstract

Faculty development programs are often used in universities and higher education institutes to develop the knowledge and skills of their teaching personnel. At the same time, these faculty development programs often remain ineffective because lecturers are not fully using what they learned in their courses, classes, and seminars. The purpose of this case study was to explore the goals higher education teachers had after professional development programs for applying newly trained knowledge and skills. Grounded in 3 × 2 achievement goal model and Ryan and Deci's (2018) goal contents theory, the study addressed the research question: Which types of transfer goals do university teachers have after teacher education? Participants were three lecturers that were selected following the procedures of the maximum variation sampling schema. Adopting a mixed methods approach, data were collected with questionnaires (aimed at profiling the goal orientations of the teachers) and reflective interviews (aimed at reconstructing the goals and goal contents teachers had for transferring the trained skills). Triangulation of the quantitative and qualitative data using the documentary method resulted in a typology of intrinsic, mixed, and extrinsic transfer goals. Directions for future research associated with instrument development and theory building as well as implications for the educational practice in human resource development programs of higher education institutes are discussed.

## 1 Introduction

Faculty development programs are often used in universities and higher education institutes to develop the knowledge and skills of their teaching personnel (Leslie et al., 2013; Postareff et al., 2007; Stes et al., 2010). At the same time, these faculty development programs often remain ineffective because participants are not fully using what they learned in their lectures, classes, and seminars (Leslie et al., 2013). It is thus a question of empirical and practical relevance to study why lecturers and teachers in higher education decide to transfer the trained lecture skills. One starting point in addressing this question is to focus on the goals associated with transfer of training activities.

### 1.1 Faculty development programs for university teachers

In higher education, faculty development programs aim to advance the knowledge and skills of university teachers. Programs typically have the purpose of developing teaching approaches, instructional strategies, course design, curriculum development, assessment practices, or teacher identity (Abu-Rish Blakeney et al., 2016; Cheng et al., 2015; Phuong et al., 2018). For example, Brinkley-Etzkorn (2018) examined the influence of faculty development training on higher education teachers' knowledge to teach online; the findings indicated only moderate improvements in the participants' teaching effectiveness and no improvements in student evaluations of teaching. Taylor and Znajda (2015) reported different patterns of findings associated with faculty development, indicating the significance of a trusted network of teaching colleagues for successful transfer of training (Froehlich and Gegenfurtner, 2019). Strong evidence suggests that faculty development programs are not always as effective as desired by universities (Brinkley-Etzkorn, 2018; Leslie et al., 2013; Saroyan and Trigwell, 2015), and the impact of faculty development is not always systematically examined (Chalmers and Gardiner, 2015; Phuong et al., 2018). Clearly, the limited impact of faculty development can be associated with the limited measurement and analysis practices employed, as for example Phuong and colleagues convincingly argue. However, one could also argue that the impact of faculty development is limited because the higher education teachers decide not to transfer their developed skills to their workplace: the lecture halls and seminar rooms in universities. If this second argument was true, then we need to focus on the motivational processes of university teachers and the goals participants tend to associate with the transfer of faculty training.

### 1.2 Associations of goals and training transfer

Goals can be defined as mental representations of desired end states (Fujita and MacGregor, 2012). Transfer of training can be described as the application of trained knowledge and skills to the workplace (Ford et al., 2018; Segers and Gegenfurtner, 2013). For decades, researchers in human resource development, applied psychology, and adult education have been interested in understanding how goals influence what adult learners do, and why they engage in these activities (e.g., Beier and Kanfer, 2009; Damşa et al., 2017; Latham, 2012; Ryan and Deci, 2018; Testers et al., 2019b). Among the many aspects associated with goal pursuit are the individual goal orientations and goal contents participants have. A closer examination of goal orientations and goal contents in the context of human resource development and training can help develop an understanding why learners are motivated to apply trained knowledge and skills to the workplace. As such, the study of the reciprocal alignments between goals and transfer contributes to the wider body of evidence on training motivation (Colquitt et al., 2000; Ford et al., 2018; Gegenfurtner et al., 2019b; Noe, 1986; Quesada-Pallarès and Gegenfurtner, 2015; Testers et al., 2015). In the psychological research literature, goals are theoretically modeled and empirically examined as goal orientations and goal contents.

Goal orientations can be defined as competence-based aims used to guide achievement behavior (Dweck and Leggett, 1988; Elliot et al., 2011; Gegenfurtner and Hagenauer, 2013). The literature on transfer of training has examined goal orientations mostly as predictor variables (e.g., Burke and Hutchins, 2007; Chiaburu and Marinova, 2005; Jacot et al., 2015; Laine and Gegenfurtner, 2013; Vanthournout et al., 2015). Vandewalle (1997) was among the first to test the influence of goal orientations on transfer in professional training contexts (Brett and Vandewalle, 1999; Vandewalle et al., 1999). Past meta-analyses identified stable correlations between goal orientations and transfer of training. For example, Blume et al. (2010) reported population correlation coefficients of 0.16 for learning goal orientation, 0.07 for prove-performance goal orientation, and -0.12 for avoid-performance goal orientation with transfer of training. Gegenfurtner (2011) reported population correlation coefficients of 0.27 for mastery orientation, 0.04 for performance orientation, and -0.11 for avoidance orientation. In the educational psychology literature, theoretical conceptualizations of goal orientations have expanded the dimensionality of the goal orientation construct. Among the recent conceptualizations is Elliot et al. (2011) 3 × 2 achievement goal model. This model specifies six different achievement goals alongside three definition (task, self, other) and two valence (approach, avoidance) components of competence. The six goal dimensions are task-approach goals (e.g., performing a task correctly), task-avoidance goals (e.g., avoiding to perform a task incorrectly), self-approach goals (e.g., performing better than oneself did in the past), self-avoidance goals (e.g., avoiding to perform worse than oneself did in the past), other-approach goals (e.g., performing better than others), and other-avoidance goals (avoiding to perform worse than others). To the best of our knowledge, the 3 × 2 achievement goal model with its six goal constructs (Elliot et al., 2011) has not yet been applied to human resource development and training contexts. It is thus an interesting question to address the goal orientation profiles of training participants in faculty development programs.

In addition to achievement goal theories, another prominent theory that addresses goal pursuit is Ryan and Deci (2018) goal contents theory. This theory developed within the framework of self-determination theory (Kasser and Ryan, 1996; Ryan and Deci, 2018) and is devoted to characterize the contents of individual life goals. These goal contents, or aspirations, can be intrinsic or extrinsic. As Ryan and Deci (2018, p. 21) note:

“Intrinsic aspirations are those goals that are rewarding in their own right, providing relatively direct satisfaction of the fundamental psychological needs for autonomy, competence, and relatedness. Examples are personal growth, meaningful relationships, and community contributions. Extrinsic aspirations, in contrast, are those built around contingent satisfactions—they make a priority of goals that are not in themselves satisfying but that may be seen as instrumental to getting unmet needs fulfilled. They include such goals as attaining wealth and material goods, acquiring fame and power, and maintaining one's attractiveness and outer image.”

To our knowledge, goal contents theory has not yet been applied to the human resource development or training context. It is thus a timely exploration to study how the motivation to transfer training resonates with intrinsic or extrinsic aspirations. For example, it seems reasonable to expect individual differences as a function of goal contents. While some participants might be interested in applying newly trained skills to grow personally and develop their skill repertoire (an intrinsic goal pursuit), other participants might be interested in applying new skills to increase their reputation as a competent performer (an extrinsic goal pursuit). More research is needed to develop hypotheses addressing which goal contents participants desire to attain after attending training programs.

### 1.3 Aims and research question

The aim of this study was, therefore, to identify the goals participants have after faculty development programs for applying newly trained knowledge and skills. Such an examination is important to better understand why faculty development programs sometimes show limited effectiveness in developing the teaching of university teachers (Brinkley-Etzkorn, 2018; Phuong et al., 2018; Saroyan and Trigwell, 2015). To the best of our knowledge, a study examining the goals of higher education teachers for transferring trained knowledge and skills from faculty development programs has not yet been performed. Grounded in the 3 × 2 achievement goal model (Elliot et al., 2011) and goal contents theory (Ryan and Deci, 2018), we were interested in reconstructing different types of transfer goals to understand the reasons underlying goal pursuit in training contexts. Specifically, we aimed to reconstruct how the participants' motivation to transfer was associated with their goal orientation profile (task-approach, task-avoidance, self-approach, self-avoidance, other-approach, other-avoidance) and their intrinsic vs. extrinsic aspirations (personal growth, community contribution, wealth, fame) as university lecturers in higher education. The research question was: Which types of transfer goals do participants have after training?

## 2 Method

### 2.1 Participants and sampling

University staff members who participated in faculty development programs of a large university in Southern Germany were invited to participate in the study. The faculty development programs were targeted at newly appointed staff members and aimed at preparing participants for their lecturing role. The multi-day programs covered relevant skills for teaching at undergraduate level, including presentation, feedback, and assessment skills. Participation in the study was voluntary (Gegenfurtner et al., 2016). Participants received no financial compensation; they were assured that all of their responses would remain confidential.

The sampling procedure for selecting the participants followed the maximum variation sampling schema (Onwuegbuzie and Leech, 2007; Yin, 2014). In this purposive sampling method, participants are carefully chosen to achieve a heterogeneous and diverse set of participants “such that all or most types of individuals, groups, or settings are selected for the inquiry” (Onwuegbuzie and Leech, 2007, p. 112). The sampling included four steps. Step 1: A one-page flyer was distributed in class to a total of 92 university staff members who participated in the faculty development programs during a semester to inform them of the study aims; if they were interested in the study, they could opt to receive a follow-up email with more detailed information on the study procedure. Step 2: From the 92 staff members, 19 participants indicated their interest and received the follow-up email. Step 3: From these 19 interested participants, 6 staff members were selected and interviewed individually. Step 4: After transcribing the interview recordings, the transcripts of three participants (1 female, 2 males; mean age: 31 years, *SD* = 5.20) were selected to be included in this study because they represent maximally heterogeneous cases with regard to their transfer goals (Onwuegbuzie and Leech, 2007; Yin, 2014). Informed consent was obtained from all participants, and ethical approval in complying with all regulations was obtained from the Technical University of Munich, Germany.

### 2.2 Data collection

Two approaches were used to identify the transfer goals of the participants: an interview and a questionnaire. The interview aimed at reconstructing the goals and goal contents participants had for transferring the trained skills. The questionnaire aimed at profiling the goal orientations of the participants.

The interviews were semi-structured and aimed at identifying the goals participants had for applying the trained skills to their own teaching. The interview guideline was identical for all participants and included three sets of questions. First, participants were asked to describe their experience in teaching and previous faculty development programs. Second, participants were asked to describe any reasons why they would transfer the newly trained teaching skills. Third, participants were asked to reflect on their life goals associated with personal growth, community contribution, wealth, and fame, with a focus on how transferring the trained skills to their own teaching would contribute to attaining these life goals. The interviews unfolded as a dialogue between the interviewee and the interviewer. Interview duration ranged from 17 to 28 min. A trained interviewer performed all interviews via phone with the participants individually. The interviews were recorded and transcribed verbatim.

After completing the interviews, participants received a link to a web-based questionnaire. The questionnaire was adapted from Elliot et al. (2011) 3 × 2 achievement goal questionnaire and consisted of 18 items that measured six dimensions of participants' achievement goals, with 3 items per dimension. These goal dimensions indicate why participants transfer the trained teaching skills to their classes. The dimensions included task-approach goals (e.g., “to lecture well”), task-avoidance goals (e.g., “to avoid lecturing poorly”), self-approach goals (e.g., “to give better lectures than before the faculty development program”), self-avoidance goals (e.g., “to avoid lecturing worse than before the faculty development program”), other-approach goals (e.g., “to give better lectures than other professors”), and other-avoidance goals (e.g., “to avoid lecturing worse than other professors”). Participants responded on a 1 (*not true at all*) to 7 (*extremely true*) Likert scale how true each statement was for them. The 3 × 2 achievement goal questionnaire has demonstrated high levels of internal consistency (Elliot et al., 2011).

### 2.3 Data analysis

Data from the questionnaire were analyzed quantitatively with means and standard deviations to profile the participants' goal orientations and to triangulate the interview data. Data from the interview transcripts were analyzed qualitatively using the documentary method (Bohnsack, 2013; Nohl, 2010). The documentary method is a method for qualitative data analysis that involves searching for patterns underlying a variety of different realizations of meaning. Following Garfinkel (1967, p. 78), utterances in the interview transcripts were treated “as the ‘document of’, as ‘pointing to’, as ‘standing on behalf of’ a presupposed underlying pattern. Not only is the underlying pattern derived from its individual documentary evidences, but the individual documentary evidences, in their turn, are interpreted on the basis of ‘what is known’ about the underlying pattern. Each is used to elaborate the other”. Following the documentary method (Bohnsack, 2013), the interview transcripts were analyzed in three steps. The first step involved the formulating interpretation. The aim was to establish the “what” of the interview text—that is, the “actual appearance” (Garfinkel, 1967, p. 78) of evidence. Specifically, the interview transcripts were segmented chronologically according to the appearance of topics and then paraphrased and condensed. The second step of the documentary method involved the reflecting interpretation. The aim was to establish the “how” of the interview text—that is, the “presupposed underlying pattern” (Garfinkel, 1967, p. 78). Specifically, the sequence of topics from step 1 was semantically interpreted and then compared across interviews to analyze the orientation frameworks within which the participants reconstructed their transfer goals. Finally, step 3 of the documentary method involved type formation. The aim was to generate different types of transfer goals in the form of common conclusions across interviews. Specifically, the different orientation frameworks identified in step 2 were abstracted from the interview transcripts and formulated as types in their own right. The interpretation of these sense-genetic types was finalized using further segments of the interview transcripts to establish and reconstruct the constellations within which the transfer goals evolved.

## 3 Results

**Figure 1:**
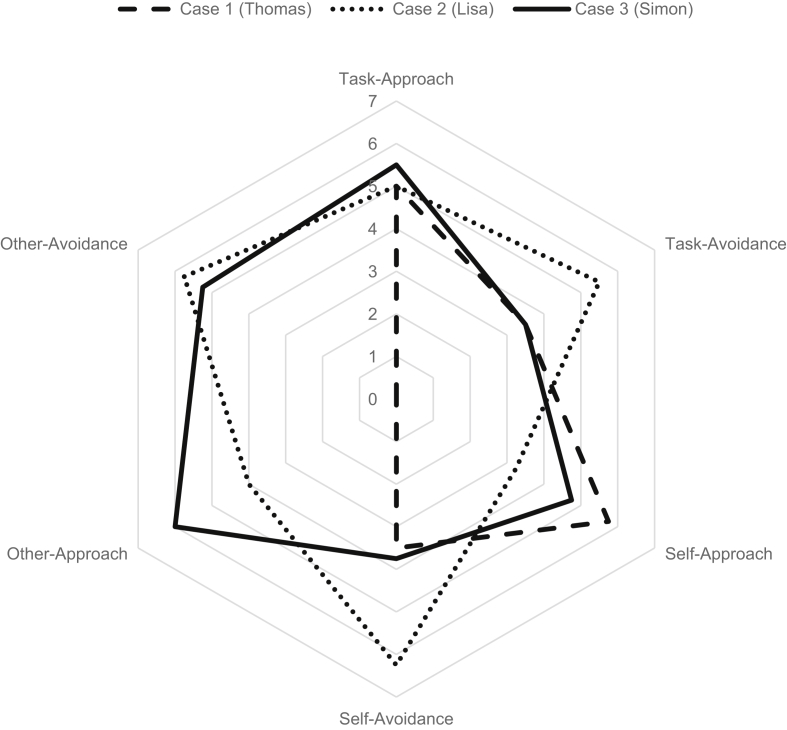
Goal orientation profiles of all cases.

This section illustrates the findings from the questionnaire and interview data analyses. Figure 1 presents the goal orientations of all three cases. The interview data are presented subsequently for each case, first with regard to the goals for transfer and then with regard to the goal contents.

### 3.1 Case 1

The first case was Thomas, a 25-year old doctoral student who works as a research assistant in the department of electrical engineering. Thomas has a self-reported teaching experience of three years. He has previously attended two other faculty development programs because, in his own words, “it is important to be a good lecturer to students and to motivate them, and also to make them interested I mean raise their interest in electrical engineering”. Figure 1 presents Thomas' goal orientation profile. He scores highly in task- approach (5.0 on a 7-point Likert scale) and self-approach (5.75) goals and has a zero score in the two other-oriented goal orientations. Thomas' profile thus represents an approach-based goal pursuit.

Among the transfer goals of Thomas was the wish to improve his own teaching for two reasons. First, he wanted to transfer the trained knowledge and skills to offer better lectures to his students. Thomas said: “Well, so, I think that students have the right to experience good teaching and then one must become better, with the help of training courses, because no one is a perfect lecturer”. Eventually, he said, “it is the students we teach for, so they should like my lectures so that they like to attend my lectures. They should become interested in the topic [electrical engineering].” Second, Thomas wanted to transfer the trained knowledge and skills to develop his own teaching personality. He said: “This is how I see it because in my opinion it would be nice to develop some time an own teaching style, a personality as a lecturer, what is also for me a very good thing to develop. So, yes, this is why I use the training too”. His transfer goals are thus associated with benefitting his students and developing an own teaching personality.

When asked about goal contents, Thomas related to intrinsic aspirations (personal growth and community contribution) rather than to extrinsic aspirations (fame, wealth). He said: “Money, mhh, does not interest me. I am a very simple guy here. Well, money is nice to have, but I don't care about it”. Similar to his neutral association with wealth, he indicated that, “becoming famous as a teacher was not among my goals, no, I did not pursue that consciously. To be honest, fame or image does not interest me at all.” These utterances correspond with Thomas' very low (zero) scores in the other-oriented goal orientations presented in Figure 1. In contrast, Thomas was very clear about his goals associated with personal growth. “Yes, definitely, this is one of my goals, absolutely, personal development, and yes, I want to develop and improve what I do as a lecturer”. Thomas' aspirations for transfer are thus strongly intrinsic.

### 3.2 Case 2

The second case was Lisa, a 34-year old post-doctoral researcher from the department of sports science. Lisa has a self-reported teaching experience of nine years. She has previously attended two other faculty development programs because, in her own words, “I think it is meaningful to learn how to deal with this group of 300 people [in the lecture hall]. I feel I am lacking the right tools”. Figure 1 presents Lisa's goal orientation profile. Similar to Thomas in Case 1, also Lisa scores highly in task-approach goals (5.0). In addition, she has high scores in all three avoidance-oriented goal orientations, including task-avoidance (5.5), self-avoidance (6.25), and other-avoidance goals (5.75). Lisa's profile thus represents an avoidance-based goal pursuit.

Among the transfer goals of Lisa was her wish to avoid doing anything wrong. She said that “I became aware in the program what I definitely do wrong all the time. I want to avoid that in the future. This is my striving for perfection… because, well, I want to have everything done properly and in orderly fashion”. Lisa said that she “does not want to give my lectures like the ones I heard as a student ten years ago, but there are now new techniques and methods and also new approaches, this is all changing strongly now I think, a bit, how lectures are. So of course then I want to give a young and modern lecture and not something a bit more dated.” Ultimately, she said, I want to get away from doing always the same”. These utterances reflect her avoidance-oriented goal orientation profile presented in Figure 1. She admits that she also has role models she aspires during transfer. “I have some certain examples because I have experienced other lecturers or some speakers in conferences who have well impressed me because of their presentation and performance.” For Lisa, transferring the trained knowledge and skills “should give me a bit more confidence.”

When asked about goal contents, Lisa related to both intrinsic and extrinsic aspirations. Concerning fame, she said: “I want my lectures to have a very high level so that they become well known.” Concerning community contribution, she admits that “this is a very altruistic question. I, no, I want to contribute to society more through my research, not so much through teaching.” Concerning personal growth, Lisa strongly confers with these aspirations. She said: “Yes, well, the way I see it is, that, if you always keep to the level you are at the moment, then you don't develop. And I thought I have to do something to avoid being worse and, well, to develop myself as a lecturer.” In her own words, “I wanted to look beyond my own nose and to leave that, how should I call it, comfort zone.” Lisa's aspirations for transfer are thus mixed, including both intrinsic and extrinsic elements.

### 3.3 Case 3

The third case was Simon, a 34-year old associate professor in the department of medicine. Simon has a self-reported teaching experience of nine years. He has previously attended three other faculty development programs. His training motivation is, in his own words, twofold: “Primarily I do it for myself and then of course also to prove a potential future employer that I can do this [give lectures]”. Figure 1 presents his goal orientation profile. Similar to the previous two cases, also Simon scores highly in task-approach goal (5.5) and has high scores in both other-oriented goal orientations, including other-approach (6.0) and other-avoidance goals (5.25). Simon's profile thus represents an other-oriented goal pursuit.

Among the transfer goals of Simon was his wish to offer alternative presentation methods “and something new, also something interactive and joyful”. He intended to professionalize his lectures. However, he admits: “It is hard to change my presentations because of time constraints”. He also said that “I don't attend every training course primarily with that goal, so, well there are topics, for these I know that I do it to expand my horizon but I can't use that one-to-one in my teaching. I think everybody has to find an own way to apply [trained skills]”.

When asked about goal contents, Simon related to extrinsic aspirations like wealth and, most strongly, to fame. Concerning wealth, he said that he does not think he benefits from improved lectures financially. “I see it more the other way round that I bring something financially and not that I get something”. Participating in the faculty development programs is thus an investment for him, something he brings financially in terms of the costs for training participation. Concerning fame, Simon said that applying the trained knowledge and skills should increase the reputation of his lectures. “In terms of lecturing, this is clearly a goal, yes. I strongly value to be well known one day and that students come to my lectures, to my teaching, and of course then they evaluate me positively”. Receiving positive ratings in student evaluations are thus an important goal for him, which resonates well with his other-oriented goal orientation profile presented in Figure 1. He said: “Well, the one thing is that, in our department, there are certain professors who always get an award for best teaching, which is a very nice status.” Receiving such an award is one of his extrinsic aspirations for transfer. “So, it does not have to be that award necessarily, but to be evaluated positively, absolutely, so that students say he is a good lecturer and to be known for lecturing. So, yes, fame, definitely.” Simon's aspiration for transfer are thus strongly extrinsic.

### 3.4 Types of transfer goals

After presenting each case individually, the next step includes a comparative mapping of the three cases to generate types of transfer goals (Bohnsack, 2013; Garfinkel, 1967; Nohl, 2010). The type formation aimed at abstracting instances of transfer goals described and clustered in the interview utterances. Type formation included two steps. In a first step, the sense-genetic type formation identified different types of why participants intended to apply the newly trained knowledge and skills. These instances are interpreted as homologous goal patterns. Table 1 presents the three types with utterances from the interview narratives.

**Table 1:** Sense-genetic type formation.

	Type 1	Type 2	Type 3
Utterance 1	“I apply the newly trained knowledge and skills”		
Utterance 2	“because I want to develop the way I teach”	“because I want to avoid errors in the way I lecture”	“because students should evaluate my lectures positively”
Utterance 3	“to offer good lectures to my students”	“to offer better lectures than the ones I attended as a student”	“to receive the award for best teaching in my department”

In a second step, the three types were further contextualized with additional documentary evidence from the transcripts and from the questionnaire. This additional evidence helped reconstruct the constellations associated with which goal orientation profile emerged; what were the dominant goal contents; what was deemphasized; what were the aspired secondary outcomes; and which factors influenced the transfer goals. Ultimately, this analysis resulted in three sociogenetic types of transfer goals presented in Table 2: (a) intrinsic transfer goals, (b) mixed transfer goals, (c) and extrinsic transfer goals.

**Table 2:** Sociogenetic types of transfer goals.

	Intrinsic transfer goal	Mixed transfer goal	Extrinsic transfer goal
What was the goal orientation profile?	Approach-orientation	Avoidance-orientation	Other-orientation
What were the dominant goal contents?	Personal growth, community contribution	Personal growth	Fame
What was deemphasized?	Other-orientation, fame, wealth	Wealth, community contribution	Community contribution
What were the aspired secondary outcomes?	Teaching personality, student interest	Perfectionism, error avoidance	Student evaluations, teaching award
Which factors influenced the transfer goals?	The student rights for good teaching	Role models	Time constraints, professors with teaching awards

The type of intrinsic transfer goal emerged in association with an approach-oriented goal orientation profile. The dominant goal contents were a pursuit for personal growth and development as well as for contributing to the student community. What was less pronounced and deliberately deemphasized was an other-orientation, wealth, or fame. Hence, the transfer goals were framed intrinsically to attain desired secondary outcomes such as the development of an own teaching personality and a nourishment of student interest in the topics that were lectured. Factors that influenced the transfer goals included a perceived responsibility for teaching well to meet the self-proclaimed rights of students to attend good lectures.

The type of mixed transfer goal emerged in association with an avoidance-goal orientation. Most strongly articulated was the aspiration for personal growth and development. Less emphasized were other (both intrinsic and extrinsic) goal contents such as wealth and community contribution. The avoidance-orientation in the goal orientation profile corresponded with the aspired secondary outcomes of transfer in the form of being able to avoid errors and achieving levels of perfectionism. The transfer goals were inspired by role models, that is: significant others, primarily lecturers, towards whom the transfer goals were oriented.

Finally, the type of extrinsic transfer goal emerged in association with an other-orientation. The most dominant goal content was fame, and transfer was framed as being instrumental to achieving the position of a lecturer well known for excellent lectures. Less emphasized was community contribution. Among the aspired secondary outcomes were positive student evaluations, which should ultimately afford awards for good teaching. Other professors who had already been awarded served as supporting factors for transfer, while time constraints were articulated to hinder extrinsic goal pursuit.

## 4 Discussion and conclusion

The purpose of the present paper was to explore the goals university teachers reconstructed after attending faculty development programs for applying newly trained knowledge and skills (Abu-Rish Blakeney et al., 2016; Brinkley-Etzkorn, 2018; Gegenfurtner and Hagenauer, 2013; Leslie et al., 2013; Postareff et al., 2007; Stes et al., 2010). To the best of our knowledge, this study is the first to examine the goals of higher education teachers for transfer. We used the theoretical frameworks of the 3 × 2 achievement goal model and goal contents theory (Ryan and Deci, 2018) to analyze why participants were willing to engage in transfer activities. The findings of the three cases illustrate different goal types. A first type of transfer goal was associated with an intrinsic goal striving, in which the transfer activities were contingent on a desire for personal growth and student benefits. The second type of transfer goal was a mixed goal striving characterized by personal growth tendencies and avoidance orientations. Finally, the third type of transfer goal was an extrinsic goal striving, driven by fame, positive student evaluations, and a strong other-orientation. Grounded in contemporary goal theories (Elliot et al., 2011; Ryan and Deci, 2018), the outcomes of this theory-driven multiple case study illuminate why participants in faculty development programs transfer trained lecture skills to their teaching activities.

This qualitative exploration of different goal types is useful because it offers a number of directions for future research on instrument development, intrapersonal examinations, and theory building. A first direction concerns the development of quantitative survey instruments for the large-scale assessment of transfer goals. The qualitative reconstruction of goal types in this study can be used as a foundation to estimate the frequency and occurrence of differential goal qualities in trainee populations. As such, this survey development can consider the external validity of the developed items when compared with other established instruments, including the aspiration index (Kasser and Ryan, 1996) and the 3 × 2 achievement goal scales (Elliot et al., 2011); furthermore, it would be conceptually relevant to test their structural interrelations and estimate the predictive validity of goal types on motivation to transfer and transfer of training (Gegenfurtner et al., 2010). A second avenue for future research concerns the use of intrapersonal approaches to identify different goal profiles and how they change over time. A number of constructs in motivation research are situative and change over time whereas some facets of human motivation remain stable (e.g. Beier and Kanfer, 2009; Bosset and Bourgeois, 2015; Jacot et al., 2015; Laine and Gegenfurtner, 2013; Testers et al., 2019a). It would be interesting to examine how stable or fluid individual transfer goals are as time after training unfolds. Finally, a third implication for future research concerns theory building to include transfer goals, goal orientations, and goal contents in the kaleidoscope of motivational variables to expand current conceptualizations of training motivation in research on human resource development (Beier and Kanfer, 2009; Colquitt et al., 2000; Elliot et al., 2011; Ford et al., 2018; Quesada-Pallarès and Gegenfurtner, 2015; Ryan and Deci, 2018) and to examine the nomological network between goal variables and other motivational predictors of training transfer, including training satisfaction, transfer interest, and the utility values of training and transfer (Ebner and Gegenfurtner, 2019; Gegenfurtner, 2019; Gegenfurtner et al., 2019a; Knogler et al., 2015).

The study also has a number of practical implications that should be noted. First, the identification of different goal profiles of prospective trainees can be used to redesign faculty development courses. Arguably, the effectiveness of training programs could be enhanced if courses are tailored to match different goal profiles. Another practical implication relates to post-training interventions. If trainees have their predominant goal profiles screened, then trainers could use these differential patterns of goal contents and goal orientations to discuss how the transfer to the trainees' teaching activities could be facilitated. Ultimately, this would further improve the training effectiveness of faculty development programs (Leslie et al., 2013; Postareff et al., 2007).

This study also has some limitations that should be noted. First, the faculty development programs were all offline face-to-face courses. The goal profiles are thus not easily generalizable across the range of training modalities, and future research can aim at replication in digital, blended, and hybrid training programs (Cheng et al., 2015; Ford et al., 2018; Gegenfurtner and Ebner, 2019; Gegenfurtner et al., 2017; Gegenfurtner et al., 2020; Siewiorek and Gegenfurtner, 2010; Testers et al., 2019b). Second, the empirical analyses centered on three case presentations. While multiple case studies are, per definition, interested in the particularities of single case instances, we shall note that future studies can aim to use larger sample sizes. Finally, the study concerned faculty development programs of teachers in higher education. Thus, the study findings must be generalized with caution to other trainee populations, including non-traditional students and employees in corporate contexts (Gegenfurtner et al., 2018; Reinhold et al., 2018; Testers et al., 2015; Vanthournout et al., 2015).

To conclude, this qualitative study reported the reconstructing of transfer goals of university faculty who participated in development programs (Stes et al., 2010). The study was innovative and explorative, largely because the 3 × 2 achievement goal model (Elliot et al., 2011) and goal contents theory (Ryan and Deci, 2018) have, to the best of our knowledge, not yet been applied in contexts of human resource development and training. The study is also innovative because it is among the very few to employ the qualitative analyses of the documentary method (Garfinkel, 1967; Nohl, 2010). Future research can use the findings presented here to further examine and deepen our understanding of the motivational dynamics associated with transfer in faculty development programs.

## Declarations

### Author contribution statement

A. Gegenfurtner: Conceived and designed the experiments; Performed the experiments; Analyzed and interpreted the data; Contributed reagents, materials, analysis tools or data; Wrote the paper.

### Funding statement

This research did not receive any specific grant from funding agencies in the public, commercial, or not-for-profit sectors.

### Competing interest statement

The authors declare no conflict of interest.

### Additional information

No additional information is available for this paper.
